# Supplementary material for: Functional information from clinically-derived drug resistant forms of the Candida glabrata Pdr1 transcription factor
Source: PLoS Genet. 2020 Aug 25;16(8):e1009005. doi: 10.1371/journal.pgen.1009005 (PMC7473514; doi:10.1371/journal.pgen.1009005)
Supplement: Attachment — Submitted filename: Response to Reviewers 6-29.docx [file pgen.1009005.s002.docx]

Dear Dr. Mitchell,

Thank you for your editorial handling of our manuscript and we thank the reviewers for their thorough consideration of our work. Below is a point-by-point response to each of the concerns raised by all three reviewers. We have included new experimental data consisting of qRT-PCR analyses of several other Pdr1 target genes as requested by the reviewers. The qRT-PCR analyses support and strengthen our conclusions relative to the function of Pdr1. CDR1 is the major transporter regulated by the Pdr1 GOF forms we have analyzed here. The Triple point mutant is the strongest PDR1 allele as evidenced by highest CDR1 expression in the absence of Med15A; the introduction of the D1082G allele into the Triple mutant (to generate the Quadruple mutant) decreases the transcriptional activity of such strain, as confirmed by two fold decrease in CDR1 mRNA levels in cells lacking Med15A. We also provide new supplemental data describing interactions of the gain-of-function (GOF) mutants described in this manuscript with previously identified trans-acting regulator of the dnaJ family: Jjj1.

We have also extensively revised the manuscript in response to issues raised by our first submission. We hope this new version will successfully address the concerns of all three reviewers.

Sincerely,

Scott Moye-Rowley

Reviewer's Responses to Questions

**Comments to the Authors:
Please note here if the review is uploaded as an attachment.**

Reviewer #1: Gain-of-function (GOF) mutations (usually caused by single nucleotide substitutions that lead to amino acid exchanges) in the transcription factor Pdr1 result in constitutive overexpression of genes encoding multidrug efflux proteins and are the major mechanism of azole resistance in the pathogenic yeast Candida glabrata. In the absence of inducing signals, wild-type Pdr1 is kept in an inactive state by a central regulatory domain (CRD) that prevents the interaction of the C-terminally located activation domain (AD) with the transcriptional apparatus. GOF mutations that relieve Pdr1 from repression are located in the CRD or in the AD. Deletion of the CRD also results in a hyperactive Pdr1, which is toxic to the cells when expressed from its own promoter because of the high Pdr1 levels achieved by autoregulation. In addition to the CRD, trans-acting negative regulators are also involved in controlling Pdr1 activity. In the present study, the authors aimed to elucidate how different GOF mutations in Pdr1 activate the transcription factor. For this purpose, they compared the effect of different GOF mutations and combinations thereof on Pdr1 activity and dependence on the mediator subunit Med15A. They found that mutations in the AD that cause constitutive activation of Pdr1 reduce the activity of the transcription factor when combined with mutations in the CRD and conclude that the AD contains different regions that interact with trans-regulatory factors and have both positive and negative regulatory functions.

This thoroughly performed, detailed study provides interesting information about the effects of different Pdr1 mutations on the activity of the transcription factor. However, I do not fully agree with some of the authors’ conclusions, and they may consider the following comments.

General comments

1) The authors found that the strongly activating D1082G GOF mutation, as well as the unexpectedly activating LWG1097AAA mutation, in the AD actually reduce the activity of hyperactive forms of Pdr1 that already contain other activating mutations (GOF mutations in or deletion of the CRD). From this observation they conclude that the C-terminal AD has both positive and negative regulatory functions. In my opinion, there is a much simpler explanation for the findings. It is reasonable to assume that the D1082G and LWG1097AAA mutations have a negative effect on the functionality of the AD but at the same time prevent its interaction with the CRD, resulting in a derepressed state. The negative effect of the AD mutations is only seen when Pdr1 is already activated by other alterations that block interaction of the AD with the CRD. This is analogous to, for example, resistance mutations in a drug target enzyme, such as Erg11. Here, mutations that confer resistance to an inhibitor (in this case azole drugs) often reduce the enzymatic activity in the absence of the inhibitor but allow sufficient residual activity in its presence. One would not argue that the corresponding region in the wild-type enzyme negatively affects enzyme activity.

**Response:** The linked positive and negative roles of the region in the activation domain (AD) containing positions 1082 and 1097 is one of the unexpected outcomes of our analyses and provides evidence that this region has a bifunctional characteristic in terms of Pdr1 function. A clear example of the negative consequences of the AD mutant D1082G is seen when comparing the Triple mutant with the Quadruple mutant in *med15A*Δ cells (Figure 3A and Figure 3B). Note that the expression of Cdr1, the most prominent transporter regulated by Pdr1 GOF forms tested in the study, is significantly better in the presence of the Triple mutant than when the Quadruple mutant provides Pdr1 function (Figure 3B). Similarly, the ability of the D1082G lesion to rescue the toxicity of the internal deletion mutant form of Pdr1 (Figure 4B) was an unpredicted behavior but valuable in making the argument that mutations in this region of the factor can have positive effects in one context (wild-type), but purely negative effects in others (internal deletion Pdr1) on the activity of Pdr1. Unlike the comparison with mutations in Erg11, any one of these single mutants alone would exhibit dramatically more activity in these assays than the wild-type factor. This is what led us to argue that, in the context of the wild-type factor, all of these point mutants define regions that normally would negatively affect Pdr1 activity. Erg11 mutations that lie within the protein chain of this enzyme typically reduce enzyme activity (See for example this analysis from Aspergillus (Warrilow, AGS et al Antimicrob Agents Chemotherap 2015 Dec; 59(12): 7771–7778.). The effect of Erg11 drug resistant mutations occurs via a very different mechanism as alteration of residues involved in azole binding prevents both the effect of the drug but also often normal enzymatic function. It is also important to keep in mind the radically different structures of proteins like enzymes, with extensive and highly evolved structures selected for proper catalysis, and the flexible transcription factors. Domains in transcription factors can be physically separated, fused to heterologous regions and still maintain their function. In general, this is not the case for enzymes.

2) The authors argue that increased Pdr1 function upon the cumulative addition of different GOF mutations indicates that the mutations affect different regulatory mechanisms (see lines 188-193). While this may be the case, it is also possible that each mutation only partially reduces the same repression mechanism and they have an additive effect. Even the Triple mutation in the CRD might still allow some interaction with the AD, explaining why it is not as toxic as the CRD deletion.

**Response:** While we favor the idea that GOF mutants act to release Pdr1 from more than one negative input, we cannot eliminate the suggestion of this reviewer that these mutations partially relieve the factor from a single common regulatory step. We have modified the sentence mentioned above (see line 202) to reflect the open nature of this question.

3) Does the fact that both the D1082G mutation and the LWG1097AAA mutation activate wild-type Pdr1 and reduce the activity of Pdr1 containing other GOF mutations prove that they define two separate regions interacting with the CRD as well as with different coactivators (see also the model in Fig. 9A)? Is it not possible that both mutations result in conformational changes that alter the interaction of the AD (at whatever sites) with the CRD and coregulators?

**Response:** We proposed this model as a means to fit all these data into a consistent framework. More data are required to actually conclusively demonstrate its various predictions that reflect specific interactions of Pdr1. However, the model as shown is consistent with the data reported here and elsewhere. Whether the specific mutations shown here interrupt binding interfaces directly or indirectly requires experiments to probe these interactions. We have used TAP-tagged versions of Pdr1 GOF forms to identify co-purifying proteins and have found different interactions of SAGA components and Mediator subunits with individual GOF forms, among other proteins. We are currently confirming these potential interactors to determine how particular GOF mutant forms of Pdr1 interact with verified co-regulators.

Other comments

4) Fig. 3A, 7B, and 8A: Why were different media used for the control plates and the fluconazole-containing plates (SC-ura or SC-leu in controls versus YPD in test plates)? Formally, the mutations might affect growth on YPD, not just in the presence of the drug.

**Response:** These mutations are all carried on plasmids and minimal medium was employed to demonstrate that, at the start of the experiment, the plasmids were present by complementation. We see the same trends if the fluconazole experiments are performed on minimal media but the level of drug that must be used is extremely high. While growth of these plasmid-containing strains on YPD can easily be done, we cannot be certain that any plasmids would be maintained.

5) Line 239: The data shown in Fig. 4B show that the presence of the D1082G mutation in Pdr1 lacking the CRD resulted in a 2-log increase, not a 3-log increase (as stated in the text), in the transformation efficiency.

**Response:** Corrected in the text.

6) Line 246: “Figure 4B” should probably read “Figure 4C”.

**Response:** Corrected in the text.

7) Line 288: “compare lanes 3 and 6” should read “compare lanes 2 and 6”. One can also refer to Fig. 5C, in which protein levels were quantified, and compare columns 3 and 5.

**Response:** Corrected in the text.

8) The legends to Figs. 2 to 8 should state that a representative western blot (out of two, see methods) is shown in each case, and the error bars should be explained (standard deviations cannot be calculated from two values).

**Response:** Added to Figs. 2- 8. Error bars refer to standard error of the mean.

9) Line 81: “...overproduction of target genes...” should better read “...overexpression of target genes...”. As it stands, this would be a gene amplification, which is not the case.

**Response:** Corrected in the text.

10) Lines 565-566: What are two “biologically independent experiments” – two independent experiments (performed on different occasions) or two biological replicates (tested in parallel)?

**Response:** Two independent experiments performed on two sets of transformants. Corrected in the text.

11) Lines 392-395: “Since PDR1 is an autoregulated gene, it is difficult to ensure that the enhanced transcription of Pdr1-regulated genes is not due to the linked increase in expression of PDR1 itself seen in these GOF mutant strains rather than increased activity of each polypeptide chain”. I have difficulties understanding this line of argumentation. If the GOF mutations did not alter the transcriptional activity of Pdr1, how would the increased expression of the mutated alleles (by autoregulation) be brought about? This potential (but disproven) scenario should be explained, e.g. did the authors hypothesize that the GOF mutations affect PDR1 transcript or Pdr1 protein stability (already excluded in their previous study, see ref. 10)? The sentence also implies that demonstrating increased activity of each Pdr1 molecule dismisses the contribution of the increased Pdr1 levels. However, it is likely that both increased Pdr1 activity (i.e. of each single Pdr1 molecule) and increased levels of the mutated transcription factor contribute to the overexpression of Pdr1 target genes.

**Response:** The fact that GOF alleles produce both more Pdr1 polypeptide and target gene expression (including that of PDR1 itself) makes it impossible to separate increased levels of Pdr1 from increased activity of each polypeptide chain. This was the motivation behind using the MET3 promoter to eliminate the autoregulatory input and clamp the expression level of each Pdr1 protein form. We fully agree with the reviewer that both level and activity of Pdr1 is important in normal regulation. The contribution of our work is to provide evidence that GOF Pdr1 polypeptides are not only overproduced but also exhibit increased activity relative to the wild-type protein. We have rewritten this section of the discussion to clarify our meaning here.

Reviewer #2: In this manuscript, Simonicova and colleagues aimed to elucidate how known gain of function (GOF) mutations in PDR1 cause hyperactivity of the protein and Candida glabrata fluconazole resistance. Numerous previous publications have established that GOF mutations in PDR1 lead to increased expression of ATP-binding cassette transporter genes including CDR1. GOF mutations also lead to higher expression of PDR1 through an autoregulatory mechanism, which drives target gene expression (Khakhina, S. et al. Mol Microbiol. 2018. 107:747). In this manuscript, the authors uncouple the effects of the GOF mutations on the levels and activity of Pdr1. Using a strain where PDR1 expression is not controlled by its endogenous promoter and therefore cannot autoregulate, the authors demonstrate that D1082G and R367W GOF mutants induce higher levels of Cdr1, while having lower levels of Pdr1 than the wild-type strain. This demonstrates that Pdr1 GOF mutations increase Pdr1 activity through additional effects beyond altering Pdr1 levels. The authors demonstrate that two different mutations in the transcriptional activation domain (D1082G mutation or LWG1097AAA mutation) substantially increase fluconazole resistance and ameliorate the toxicity of a hyperactive Pdr1 mutant that has its central regulatory domain (CRD) deleted. In addition, the authors show that many of the GOF mutants have effects that are dependent on the known Pdr1 co-activator, Mediator protein Med15A, with the D1082G mutation causing increased occupancy of Med15A at the Cdr1 promoter.

Overall, the writing of this manuscript lacked accuracy and precision, causing many of the authors conclusions to be communicated in ways that are misleading or incorrect. Substantial editing of the manuscript text should be made to strengthen the paper. A limited set of novel mechanistic insights were achieved in this manuscript, but the majority of effects seen were a result of altered Prdr1 levels. Specific comments to improve the manuscript are provided below.

Specific points:
1. Controlling PDR1 expression with the non-endogenous promoter MET3 is sufficient to cause increased resistance to fluconazole (Figure 2A). This is likely due to higher expression of the Pdr1 protein, as demonstrated in Figure 2B and 2C. It would be helpful for the authors to comment on this finding in the text. In addition, the authors could provide insight into why the MET3 promoter increases the Pdr1 protein levels by almost 15-fold but the increase in activity measured by Cdr1 levels is only two-fold.

**Response:** We have inserted a line mentioning the increased fluconazole resistance of the MET3-PDR1 fusion gene in the Results section (line 159: expressing wild-type PDR1 from the MET3 promoter…). We also inserted a line mentioning a likely reason for this difference in line 173 (this shows that wild-type Pdr1 is still under…).

2. Most of the conclusions in the paper are based on quantifications of western blots, but these experiments could be strengthened significantly by statistical analyses. For example, is the increase in Pdr1 protein levels in the GOF mutants seen in Figure 2B and 2C statistically significant? Are the differences in Cdr1 levels between the Triple mutant and the Quadruple mutant in Figure 3C (described on lines 218-221) statistically significant? It also seems that error bars are not always visible (ex. Figure 8C, error bars of the Pdr1 levels are only visible for one strain) and there is no description of what the error bars demonstrate (standard deviation or standard error, how many biological replicates). If statistics cannot be done on the western blots, quantitative RT-PCR could be performed to monitor the expression of CDR1.

**Response:** We have corrected the presentations of all the western blots with the inclusion of error bars inclusion corresponding to standard error of the mean. Additionally, as suggested here, we have also included new qRT-PCR measurements of mRNA levels for CDR1 and three other Pdr1 target genes (CDR2, SNQ2, YBT1).

3. It would be useful to include the data not shown on the protein levels for Prd1 and Cdr1 for the other GOF mutants in the CRD domain or included as a Supplementary figure (line 212-213, Figure 3).

**Response:** Based on our previous analyses of the GOF mutants in the CRD region (<https://www.ncbi.nlm.nih.gov/pmc/articles/PMC5842128/> ), we selected R376W PDR1 as a representative to reduce the amount of characterization required. However, we now include functional analyses of these other CRD mutants as new Figure 3B by qRT-PCR.

4. Some of the conclusions about Figure 3 are misleading. On lines 202- 204 and 414-415 the authors indicate that the Quadruple mutant showed less fluconazole resistance than the D1082G Pdr1 at 10 µg/mL of fluconazole but all of the mutants show a similar resistance to fluconazole in the pdr1Δ strain. The statement is only true in the pdr1Δ med15AΔ strain. On lines 204-205 and lines 410-411 the authors indicate that the Triple mutant is the strongest GOF allele, but again this is only true in the pdr1Δ med15AΔ strain. These statements should be rewritten for accuracy.

**Response:** Along with our inclusion of the qRT-PCR data that show the highest Cdr1 expression in Triple mutant in the absence of Med15A among all tested strains in Figure 3B, we have rewritten the above-mentioned lines to emphasize the use of the med15A null strain to distinguish between effects of the various GOF alleles.

5. In the discussion of Figure 5A, the authors do not comment on the intriguing finding that the D1082G mutation ameliorates the toxicity of the Δ255-968 PDR1 when the wild-type PDR1 is repressed with met. This is confounded by the fact that the D1082G mutant causes lower levels of Pdr1 than the Δ255-968 PDR1 alone (also seen in Figure 6). The authors should comment on why the D1082G would affect Δ255-968 Pdr1 protein levels.

**Response:** We added a comment on why D1082G mutation reduces the toxicity of the Δ255-968 PDR1 in *pdr1*Δ cells in the discussion and agree with this reviewer that the rescue of the Δ255-968 Pdr1 lethality by the D1082G mutation was quite surprising. The effect of the D1082G on levels of the Δ255-968 Pdr1 protein is unknown and could simply be an effect of protein stability. The reason for lower levels of D1082G Δ255-968 Pdr1 protein between figures 5 and 6 must be interpreted cautiously as its production in these two figures is from two different promoters: PDR1 in Figure 5 and MET3 in Figure 6.

6. The authors indicate that the D1082G mutation reduces the function of the Δ255-968 PDR1, as it is less effective at supporting the fluconazole resistance (lines 304-307). This seems misleading, as the difference between the two mutants is very minor and is only seen in the absence of Med15A. At most, this could be interpreted as there being a minor increase in dependence on Med15A. The authors also conclude that the D1082G mutation reduces the capacity of the Δ255-968 PDR1 to induce Cdr1 levels (lines 313-316, 440-442), which again is only seen in the absence of Med15A and is confounded by the decrease in Pdr1 levels seen. These conclusions should be softened and the limitations addressed.

**Response:** We have rewritten the lines 304-307 and 313-316 for more accuracy. It is true that the effect in the MET3 system is modest, as the reviewer points out, but along with data from Figure 4 and Figure 5 this is another independent piece of data that agrees with a reduction in function caused by the D1082G mutant in the Δ255-968 Pdr1 context.

7. In Figure 7C and D, the authors compare the levels of Pdr1 and Cdr1 across 5 different western blots. It is not appropriate to compare quantification of western blot bands that were not run at the same time, on the same gel, or have been spliced and therefore we cannot interpret the results. These western blots and quantification should be repeated appropriately.

**Response:** All these data are from one single western blot. Additional lanes of data were not used and the image is spliced digitally. Space is left to emphasize this point.

8. In lines 375-377, the authors describe the double mutant as being highly defective in expression of both Pdr1 and Cdr1. This is not true, as Pdr1 levels in the double mutant are similar to the wild-type strain or the AAA single mutant. The double mutant has a substantial decrease in Cdr1 levels compared to the D1082G mutant and a modest decrease compared to the AAA mutant. The wording of this statement should be corrected.

**Response:** This sentence has been revised as requested.

9. In many instances in the paper, the authors imply novel findings that Pdr1 interacts with multiple co-activators. For example, lines 116 to 118 implies that the authors provide genetic evidence that identifies a suite of coactivators that interact with Pdr1, which is not true. The authors state in the abstract that they provide genetic evidence for an element within the transactivation domain (TAD) that mediates the interaction of Pdr1 with coactivators. This is misleading, as it was only found that the D1082G mutation affects Med15 recruitment to the CDR1 promoter. These findings should be written explicitly.

**Response:** Our conclusion that multiple co-activators interact with the C-terminus of Pdr1 is based on three observations. First, loss of Med15A, shown by several labs to be required for normal wild-type Pdr1 transcriptional activation, lowers but does not eliminate the azole resistance and Pdr1-dependent activation of CDR1 gene expression in many GOF mutants tested in the study (Figure 3). Second, a *med15A*Δ strain can tolerate the presence of the Δ255-968 PDR1 allele in comparison to *pdr1*Δ strain as we have shown in our previous study (Khakhina, S. et al. Mol Microbiol. 2018. 107:747) and there is still elevated fluconazole resistance and Cdr1 expression when Δ255-968 PDR1 allele is expressed in *med15A*Δ background (Figure 7). Third, the D1082G LWG1097AAA Pdr1 in a *med15A*Δ strain produced the lowest level of fluconazole resistance and CDR1 expression (Figure 8). Together, these data form the argument that multiple coactivators must interact in a manner that requires the intact Pdr1 C-terminus. Clearly, Med15A is not the only coactivator as we can increase the defect seen in its absence by further altering the Pdr1 C-terminal TAD.

10. The manuscript could be substantially strengthened by efforts to assess the effect of the genetic alterations on interactions with the other known coactivators Bem1 and Jij1 or identifying new coactivators.

**Response:** This is likely a nomenclature issue but as far as the data from our lab and others show, proteins such as Bre5 or Jjj1 do not represent coactivators but rather repressors (Paul, S. et al Mol Microbiol. 2018 Oct;110(2):309-323; Whaley, SG. et al mSphere 2018 Feb 21;3(1):e00466-17.). Certainly, by genetic criteria, loss of either Bre5 or Jjj1 caused increased wild type Pdr1-dependent activation. This is a classic demonstration of the negative activity of a regulatory factor. We are including supplementary data in which we examine the interaction of Jjj1 with the GOF alleles tested. We provided these data only for the purposes of review as to properly characterize the interaction of Jjj1 with Pdr1 and GOF derivatives will require extensive further experimental effort. These studies are on-going but we wanted to demonstrate our efforts to address the issues raised by each reviewer. One challenge with characterization of the GOF forms of Pdr1 is that their phenotypes (fluconazole resistance and CDR1 transcription) are so strong, that mutants known to increase Pdr1 function (like *jjj1*Δ) are difficult to assess. GOF PDR1 alleles already have such strong phenotype that increases are hard to measure. We are testing Jjj1 association with these mutant Pdr1 proteins and the effect of Jjj1 overproduction but this is beyond the scope of this work.

***11. The paper could be strengthened if the authors showed the effects of alterations in Pdr1 on the expression of additional Pdr1 target genes besides CDR1 that are known to affect fluconazole efflux, such as CDR2 and SNQ2. This is integral, as previous studies have found that some GOF mutations do not alter expression of CDR1, but cause upregulation of SNQ2 (Torelli, R. et al. Mol. Microbiol. 2008. 68:186). Alternatively, the authors could perform functional assays to monitor the effect on drug efflux.

**Response:** We have included data in Figure 3B that examine CDR1, CDR2, SNQ2 and YBT1 mRNA levels. CDR1 transcript levels are the most highly induced of any of these genes by all GOF alleles analyzed.

12. It is appreciated that the system is very complex and multifaceted, but a more thorough explanation of different models of how the GOF mutations could be affecting Pdr1 activity should be presented. How do the authors explain the finding that the three single GOF mutations in the CRD are Med15A dependent but the Triple mutant is not? A discussion should be provided to explain how the repression of the CRD occurs via interactions between the TAD, as suggested in the figure legend, but is still affected by Med15A. Further discussion should be provided about the connection between the D1082G mutant and the LWG1097AAA mutant. For example, why is the increased association of Med15A with the CDR1 promoter seen in the D1082G mutant lost upon the additional LWG1097AAA mutation.

**Response:** We tried to focus our discussion on the major findings from our work. It seems that our GOF mutants do not lack Med15A-dependence but rather their dependence is greater than that of the Triple Pdr1. We are keen to understand points raised by the reviewer in the future. We are currently characterizing proteins that co-purify with Pdr1 GOF forms in hopes of finding the other coactivators that we provide genetic evidence for their role in Pdr1-dependent transcription. The loss of Med15A recruitment when combining the D1082G allele with the LWG1097AAA mutation is provocative but we cannot explain this currently. As indicated in the results section, the area around LWG1097 site (9aa TAD element) might be the primary binding site for Med15A on Pdr1 and that is why we are losing the interaction of Med15A with Pdr1-D1082G-AAA on CDR1 promoter. Compared to Pdr1-D1082G situation. Since Med15A is a member of the Tail subcomplex of Mediator, another Tail component might interact within the the activation domain of Pdr1. Loss of the binding site D1082 or LWG1097 could weaken Tail binding but loss of both would eliminate this interaction. Experiments testing this idea are underway but at an early stage.

13. The discussion section of the paper is lacking any discussion of the implications of the findings or how they expand on findings in the field. The authors could include a discussion of the known effects of Pdr1 GOF mutations on virulence and fitness of C. glabrata in animal models (Ferrari, S. PLoS Pathogens. 2009. 5:e1000268).

**Response:** The 4 single GOF alleles we characterized here were based on the mutations recovered in the Ferrari et al manuscript mentioned above in which these authors comprehensively examined the question of their virulence. Our data aim to advance the understanding of the molecular basis of how Pdr1 is regulated in these GOF alleles. Our focus was to characterize areas in the activation domain that may serve as binding sites for coactivators other than Med15A and potential therapeutic targets. We additionally included this information into the discussion section. We are currently identifying and characterizing such regulators.

Minor points
- Line 32 – amino acids.

**Response:** Corrected in text.

- Line 45 – unclear wording “define nonidentical negative inputs”, since GOF mutations give a gain of function and not a negative input.

**Response:** GOF mutations block negative regulation that affects Pdr1 transcriptional activation. The sentence was edited in the text for more clarity.

- Line 91 – unclear wording for “is a toxic transactivator”.

**Response:** Revised in text.

- Lines 99-101 – name the two transactivating negative regulators, Bem1 and Jij1.

**Response:** We assume this is a typographical error as the two trans-acting negative regulators are Bre5 and Jjj1. These are listed as requested.

- Line 148 – D1082G.

**Response:** Corrected in text.

- Line 168 – what is it meant by “specific activity”.

**Response:** This is a phrase meant to indicate the activity/quantity as in an enzyme. Each GOF Pdr1 polypeptide has more transcriptional activity than each wild-type Pdr1 polypeptide.

- Line 180 – Remove the and.

**Response:** Revised as requested.

- The legend is missing in Figure 3C.

**Response:** Figure 3 has been revised.

- Lines 217-218 – It seems misleading to say that the levels of Pdr1 were similar across all mutants when there is almost a two-fold difference in levels between the D1082G mutant and the Triple mutant.

**Response:** This section of the text has been revised.

- Lines 233-235 – include a reference to Figure 4A, which is currently missing.

**Response:** Corrected in the text.

- Line 239 – it appears to be a 2-log difference not a 3-log difference, as described.

**Response:** Corrected in the text.

- The reference to Figure 4B should be removed from line 246, since the figure does not contain data with the strain of the Δ255-968 mutant of Pdr1 with a chromosomal copy of PDR1.

**Response:** This reference should be to figure 4C and this has been corrected.

- Figure 4 D and E would be easier to interpret if the western and the bar graphs were plotted with the strains in the same order.

**Response:** Revised as requested.

- Lines 270-272 – the description of the TAP tag should be moved to Figure 4 where the TAP tagged mutants are first used.

**Response:** Moved as requested.

- Lines 286-287 (and various places) – Unclear wording of internally deleted Pdr1. Refer to it by the domain deletion or something more descriptive.

**Response:** We have corrected the wording to internal deletion Pdr1. Δ255-968 Pdr1 is now mentioned to be reffered to as internal deletion Pdr1 in the text in the line 258.

- Line 288 – the relevant comparison should be 2 and 6, not 3 and 6.

**Response:** Corrected in the text.

- Line 339-340 – the lower level of drug resistance of the LWG1097AAA mutation compared to the Δ255-968 Pdr1 alone can only be assessed in the absence of Med15A. This should be clarified.

**Response:** Clarified in the text as requested.

- A reference to Figure 8D needs to be added somewhere in the text.

**Response:** Added to the text.

- Line 423 – lesion should be changed to mutation.

**Response:** Revised as requested.

- Line 489 – C. glabrata should be italicized.

**Response:** Corrected in the text.

- Line 775 – amino acids.

**Response:** Corrected in the text.

- The references should be formatted properly so that all species names are italicized.

**Response:** Corrected in the text.

Reviewer #3

Synopsis

In this work, the Moye-Rowley group used a genetic approach to uncover possible

mechanisms governing the transcriptional control by Pdr1 in the human fungal pathogen C. glabrata. Pdr1 is the master regulator of the Cdr1 drug efflux pump, which in the case of C. glabrata, represents a prime cause of the intrinsic clinical azole resistance displayed by C. glabrata laboratory strains as well as clinical isolates. The authors use mutational approaches and reporter assays to show that mutations in two main internal domains of Pdr1 can explain and thus account for the complex regulatory roles. They provide evidence that Pdr1 communicates with various other co-activators, and engages the Med15 mediator for driving transcriptional regulation. Interestingly, the author provide genetic evidence for mechanisms through which Pdr1 can exert both positive and negative regulatory effect on target genes. Finally, the authors propose an appealing model about molecular mechanisms Pdr1 exploits for the transcriptional control of target genes.

Taken together, this is a solid piece of work addressing a topic medical relevance. The manuscript is very well written and easy to follow. The data presentation is very good, and the main conclusions are well supported by the available data. There are a few minor issues that require attention before publication can commence as outlined below.

Minor issues

A. Figure 1 should be moved into the Supplemental information, since it is of little

informational value

**Response:** We prefer to leave Figure 1 in the main part of the manuscript as it provides the geographic layout of the location of the different mutations used in this study.

B. It would be interesting to include other azole drugs for the drug resistance phenotyping of various Pdr1 constructs to test how these Pdr1 variants can be activated by other azoles.

Have the authors tested other PDR drug substrates?

**Response:** We have tested voriconazole but did not see any significant differences in resistance phenotypes (other than voriconazole requiring lower drug concentrations than fluconazole). Here we use fluconazole as this is the most commonly used antifungal drug. The reviewer raises an interesting point as our experiments with GOF mutants have not provided strong evidence that azole drugs can stimulate their function as is seen for the wild-type protein. The relative strength of GOF mutants is so high that further stimulation (such as drug induction) is difficult to assess.

C. Previous publications by the group have identified several Pdr1 targets operating in the DNA repair pathway, other ABC transporters such as Ybt1, as well as genes implicated in mitochondrial function. Have the authors tested the impact of the present Pdr1 constructs on a possible differential regulation of selected key target genes from these pathways?

**Response:** We have included qRT-PCR examining YBT1 expression profile in all GOF mutants along with two additional ABC transporter-encoding genes (CDR2 and SNQ2) in Figure 3B. CDR1 is the most responsive gene of all those tested and there is no obvious gene-specific effect of the alleles here.

D. The regulatory model is very appealing and may hold a wider applicability for binuclear Zn-finger regulators. Is the "conserved" regulatory motif present (only?) in Gcn4 or is it also present in related fungal binuclear zinc-finger regulators (including those having narrow rather than broad target gene sets)?

**Response:** This is a tough question to answer as our picture of how Zn cluster-containing transcription factors is surprisingly limited. The best understood factors of this class come from S. cerevisiae like Gal4, Leu3 and others. The only somewhat conserved domain shared between these proteins (other than the defining DNA-binding domain) would be the presence of the CRD. While there is limited sequence conservation across these different factors that allowed this CRD to be recognized some time ago (<https://pubmed.ncbi.nlm.nih.gov/9031633/> ), clear indication that conserved structural domains exist is still lacking.

E. The Discussion is somewhat centered and focused on the Pdr1 data presented in this manuscript. It would be worthwhile if the authors could expand the discussion the model in a broader context relating to other transcriptional regulators.

**Response:** Our purpose here is to provide new information about the different mechanisms of action of GOF alleles of PDR1. As mentioned above, it is difficult to extrapolate what is true for Pdr1 to other, even closely related, factors. An example of this difficulty comes from comparing our work on S. cerevisiae Pdr1 and Pdr3 with the very closely related C. glabrata Pdr1. Trans-acting regulators like Bre5 that do act on the C. glabrata Pdr1 have no role in the action of the highly conserved S. cerevisiae cognate factors. As mentioned above, we have restricted our discussion to a focus on what our data reveal about the action and control of C. glabrata Pdr1.
