# Supplementary material for: Functional information from clinically-derived drug resistant forms of the Candida glabrata Pdr1 transcription factor
Source: PLoS Genet. 2020 Aug 25;16(8):e1009005. doi: 10.1371/journal.pgen.1009005 (PMC7473514; doi:10.1371/journal.pgen.1009005)
Supplement: Attachment — Submitted filename: Response to reviewers_7-21-20.docx [file pgen.1009005.s003.docx]

1.  Interpretation of the data in Figure 3 - softening the conclusion in light of the reviewer's comments seems appropriate.

**Response:** Changed in text.

2.  Possible inconsistency of Figure 5B and text line 304 - please make sure that the experiment and reagents are described accurately and consistently.

**Response:** This has been reviewed and corrected.

3.  Some minor editorial issues, as pointed out by the reviewers.

**Response:** Please see below for a point-by-point response to the issues raised by each reviewer.

Reviewer #1: The authors have discussed and addressed my previous comments in their response letter and revised manuscript (I had not suggested additional experiments). I still think that my explanation for the fact that activating mutations in the TAD decrease the activity of Pdr1 forms that are already hyperactivated by a CRD mutation is simpler and straightforward (previous comment 1; I had compared this with resistance mutations in Erg11 as an analogy for ease of understanding, but this was not meant as a mechanistic comparison of enzymes and transcription factors). However, this is up to the authors, and readers can interpret the data for themselves.

I found some minor errors that should be corrected:

1) Line 23: “Figure 3B and C” should read “Figure 3C and D”

**Response:** Changed in text.

2) There appears to be an error in Fig. 5B and the corresponding text (line 304) that had escaped me in the original version. If I understood the experiment correctly, an antibody against wild-type Pdr1 was used here, not an anti-TAP antibody, because the latter would not detect the longer wild-type Pdr1 produced by PDR1 expression from the MET3 promoter. This is correctly described in the legend to Fig. 5B, but the labeling of the corresponding band in Fig. 5B is also wrong (delete TAP). Or did the wild-type PDR1 expressed from the MET3 promoter also contain the TAP tag (see lines 288-292)? In that case, “MET3-PDR1” should read “MET3-TAP-PDR1” in the upper left part of Fig. 5B (the use or not of italics in the designations was confusing to me) and the description in the legend be corrected. The authors should probably check the whole manuscript for the correct description of the use of anti-Pdr1 or anti-TAP antibodies.

**Response:** The reviewer is correct and this labeling issue has been corrected and checked for both Figure 5 and text. In Figure 5, Pdr1 is expressed as a fusion protein with TAP. The confusing labeling has also been fixed. We now refer to MET3 and PDR1 as genetic designations (in italics) while leaving the protein designator (TAP) in plain text.

Reviewer #2: In this revised version of the manuscript, Simonicova and Moye-Rowley have substantially improved their submission which characterizes the effect of Gain of Function mutations in the transcription factor Pdr1 on Candida glabrata fluconazole resistance.
The authors have dramatically improved the writing and clarity of the text. They have added additional information where necessary and have added additional experimental data as recommended, including the RT-PCR of CDR1 levels for the additional GOF mutants and the RT-PCR of other PDR1 target genes.
While the majority of major concerns have been addressed, some issues still remain. The biggest issue is with the interpretation of the data in Figure 3. The authors conclude that the introduction of the D1082G allele into the Triple mutant (to generate the Quadruple mutant) decreases the transcriptional activity of the strain (lines 438-439). They claim on Lines 237 to 238 that “the Triple mutant drove the highest level of Cdr1 expression, an effect that was reversed by introduction of the D1082G allele in the Quadruple mutant.”
This reviewer does not agree with these conclusions. While there is a 50% decrease in the expression levels of CDR1 by RT-PCR in the Quadruple mutant versus the Triple mutant, this is only seen in the absence of Med15A. Additionally, this 50% decrease in CDR1 levels is not reflected at the protein level in the absence of Med15A (in Figure 3C and D), suggesting that it is unlikely that these small transcriptional differences are causing the increased fluconazole sensitivity of the Quadruple mutant observed in A.
In the presence of Med15A, the addition of the D1082G mutation has no effect on the CDR1 levels compared to the Triple mutant. While there is a slight difference in Cdr1 protein levels in the Western blot between the Triple mutant and Quadruple mutant in Figure 3C and D, this effect appears minor and with the high levels of error and lack of statistical significance testing, this reviewer does not feel confident in that conclusion either. It is very clear that adding the D1082G mutation to the Triple mutant increases the fluconazole sensitivity of C. glabrata in the absence of Med15A, which the authors can emphasize in the text, but it seems misleading to conclude that this is due to changes in CDR1.
This reviewer appreciates that they tried to strengthen their point by including the quantitative RT-PCR data for the three other Pdr1 target genes. As the level of expression for the additional target genes is low and they do not show the same exact patterns as the CDR1 levels, they do not help to strengthen the authors’ claims on this point.

**Response:** We make the conclusion that the D1082 position acts to regulate Pdr1 transactivation in a positive manner based only in part on the data in figure 3. The Triple mutant produced 50% more CDR1 mRNA by RT-qPCR in Figure 3B. This can only be seen in a med15AΔ cells owing to the high level of transcriptional activation supported by all GOF alleles when Med15A is present. Second, D1082G rescues the lethality of the toxically active Δ255-968 Pdr1 transactivator. Other means of rescue of this lethal phenotype include placing the Δ255-968 Pdr1 over a wild-type copy of PDR1, using the med15AΔ genetic background, introducing a frameshift mutation into this gene or removing the capacity for PDR1 autoregulation (Khakhina, et al Mol Micro 2018). Third, when expressed in the presence of a repressible wild-type form of Pdr1 (Figure 5B and C), the Δ255-968 Pdr1 drives at least 4 times more Cdr1 expression compared to Δ255-968 D1082G Pdr1 (taking into account the 2-fold higher expression of the Δ255-968 Pdr1 form). Fourth, expression of the Δ255-968 Pdr1 and the Δ255-968 D1082G Pdr1 forms from the MET3 promoter demonstrated that Δ255-968 Pdr1 drove more Cdr1 expression and had slightly elevated fluconazole tolerance (Figure 6). Again, these effects can only be appreciated in the absence of Med15A as their transcriptional activation of target genes is extremely high. Fifth, combining D1082G with LWG1097AAA led to production of such a defective form of Δ255-968 Pdr1 that the resulting mutant protein has fluconazole resistance lowered to near background levels when Med15A is removed. We agree that one single piece of evidence is insufficient to argue for the dual functional nature of D1082 in Pdr1, but the combination of all these different assays are most simply interpreted as we have here.

We have further qualified our language (lines 238-240) in the analysis of these data but would respectfully like to point out that we reach no conclusions about D1082 here but only select this amino acid position for further study. Additionally, we never conclude the phenotypic effects are due to changes in CDR1 transcription and only use this gene as an indicator of the effects of various PDR1 mutants.

There are additional minor Comments to improve the manuscript:

- Line 32- “amino acids”

**Response:** Changed in text.

- In Figure 3B caption, it would be useful to mention that cDNA synthesis was performed and DNA is normalized to TEF1

**Response:** These experimental details were additionally included in the legend to the Figure 3B and are included in the Methods section, as well.

- Line 230 – should refer to Figure 3C and D

**Response:** Changed in text.

- Lines 232-233 – It is unclear why the authors are focusing on the fact that in the R376W Cdr1 expression is highly dependent on the presence of Med15A, when the expression of Cdr1 is highly dependent on Med15A in all the mu as noted in the text.

**Response:** This section has been revised.

- Line 275 – It should be written more clearly what the Cdr1 levels are being compared to. Is it a comparison of in the presence of PDR1 versus the absence of PDR1 for the D1082G mutant or is in the comparison of the D1082G mutant compared to the delta255-968 mutant?

**Response:** Rewritten for clarity

- Line 276 – The authors could say in Figure 4C instead of above.

**Response:** Changed in text.

- Lines 333 -335 - The authors should acknowledge that the levels of Pdr1 decrease in Figure 6B when D1082G is added to the Δ255-968 mutant, as it could be contributing to the decrease in Cdr1 levels.

**Response:** Changed in text.

- Line 337 – This is only true in the absence of Med15A.

**Response:** Text inserted at line 341 to clarify this point.

- Lines 362-366 – The strain has 3 modifications to Pdr1, not 2, as it is also a deletion of 255-968.

**Response:** Changed in text.

- Figure 7D – add a label for the Cdr1 quantification graph

**Response:** Corrected in Figure 7

- Lines 417-419 – The authors should clarify that they are referring to the D1082G and R376W mutants and the data is shown in Figure 2. In all areas of the discussion, it would increase clarity if the authors would mention which figures they are referring to.

**Response:** Corrected in text.

Reviewer #3: The authors undertook laudable efforts to address all points raised in my report on the original submission. Further, the authors added new experimental data and introduced extensive revisions, most of which were requested by the other reviewers.
While I still disagree with the response to the minor point A, it is acceptable to leave Figure 1 in the main manuscript, given that it adheres to the author guidelines of the journal.
Overall, this is a nice piece of work, offering new and interesting mechanistic insights about the function of the Pdr1 transcription factor and its cross-talk with Med15. This reviewer therefore looks forward to seeing this manuscript published in PLoS Genetics.

**Response:** We thank reviewer 3 for their generous comments.
